# Using a Smartphone Application for the Accurate and Rapid Diagnosis of Acute Anterior Intracranial Arterial Occlusion: Usability Study

**DOI:** 10.2196/28192

**Published:** 2021-08-27

**Authors:** Teppei Komatsu, Kenichiro Sakai, Yasuyuki Iguchi, Hiroyuki Takao, Toshihiro Ishibashi, Yuichi Murayama

**Affiliations:** 1 Department of Neurology The Jikei University School of Medicine Tokyo Japan; 2 Department of Neurosurgery The Jikei University School of Medicine Tokyo Japan

**Keywords:** stroke, infarction, teleradiology, smartphone, telehealth, reperfusion, neurology, mHealth, application, mobile health, mobile applications, diagnosis, diagnostics

## Abstract

**Background:**

Telestroke has developed rapidly as an assessment tool for patients eligible for reperfusion therapy.

**Objective:**

To investigate whether vascular neurologists can diagnose intracranial large vessel occlusion (LVO) as quickly and accurately using a smartphone application compared to a hospital-based desktop PC monitor.

**Methods:**

We retrospectively enrolled 108 consecutive patients with acute ischemic stroke in the middle cerebral artery territory who underwent magnetic resonance imaging (MRI) within 24 hours of their stroke onset. Two vascular neurologists, blinded to all clinical information, independently evaluated magnetic resonance angiography and fluid-attenuated inversion recovery images for the presence or absence of LVO in the internal carotid artery and middle cerebral artery (M1, M2, or M3) on both a smartphone application (Smartphone-LVO) and a hospital-based desktop PC monitor (PC-LVO). To evaluate the accuracy of an arterial occlusion diagnosis, interdevice variability between Smartphone-LVO and PC-LVO was analyzed using κ statistics, and image interpretation time was compared between Smartphone-LVO and PC-LVO.

**Results:**

There was broad agreement between Smartphone-LVO and PC-LVO evaluations regarding the presence or absence of arterial occlusion (Reader 1: κ=0.94; *P*<.001 vs Reader 2: κ=0.89; *P*<.001), and interpretation times were similar between Smartphone-LVO and PC-LVO.

**Conclusions:**

The results indicate the evaluation of neuroimages using a smartphone application can provide an accurate and timely diagnosis of anterior intracranial arterial occlusion that can be shared immediately with members of the stroke team to support the management of patients with hyperacute ischemic stroke.

## Introduction

In acute stroke management, early initiation is the most important factor in successful reperfusion therapy for both intravenous tissue plasminogen activator (IV-tPA) and mechanical thrombectomy (MT) [1,2] because the patient loses 1.9 million neurons every minute that a stroke is untreated [3]. The selection of appropriate candidates for reperfusion therapy requires neurological evaluation and neuroimaging studies to be obtained immediately after admission. Accurate diagnostic information must be shared promptly within the multidisciplinary stroke team (comprising of emergency physicians, vascular neurologists, neuroradiologists, neurosurgeons, anesthetists, and ward nurses) to progress the decision-making process.

**Figure 1:**
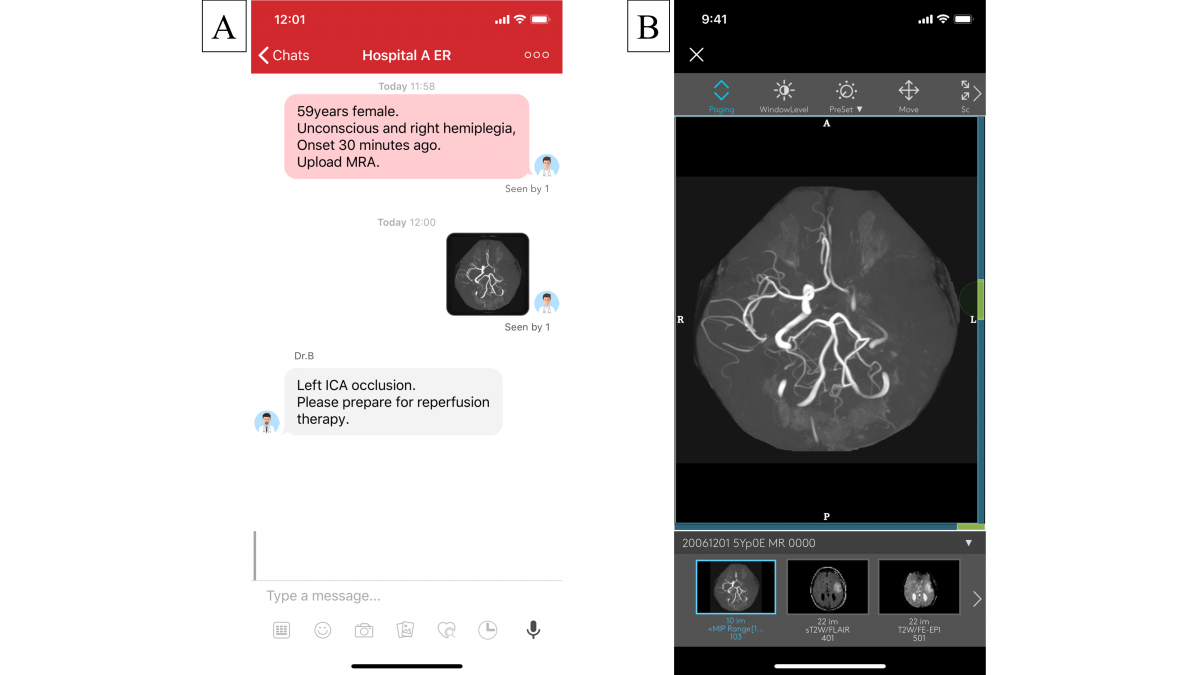
A. The JOIN smartphone application utilizes the easy-to-use interface of the social networking communication environment. B. The JOIN smartphone application displays diagnostic medical images, such as magnetic resonance imaging. ICA: internal carotid artery; MRA: magnetic resonance angiography.

Because “time is brain” in acute stroke treatment, the appropriate use of information and communication technology (ICT), particularly telemedicine for stroke or “telestroke,” has become a valuable method for reaching out to patients who are eligible for IV-tPA and MT administration. Telestroke is continually undergoing development, specifically in the United States and the European Union. The JOIN smartphone application enables the multidisciplinary stroke team to share clinical information and imaging data securely. Through JOIN, the intrahospital network system sends medical images from the hospital server to a shared chat room accessible to stroke team members only, similar to modified teleradiology and teleconferencing (Figure 1). Over 200 medical institutions in Japan and more than 11 other countries, including the United States, Germany, and Brazil, use JOIN. It is complementary to the telestroke system, particularly as an image evaluation tool [4]. In addition, the COVID-19 epidemic has increased the importance of teleradiology.

Because the evaluation of large vessel occlusions (LVO) is vital for successful MT [5-7], the accurate and rapid diagnosis of LVO is clinically relevant. ICT can play an essential role in this regard by providing intranetwork and internetwork support. At our stroke center, the patients with acute ischemic stroke are initially examined via magnetic resonance imaging (MRI) rather than computed tomography (CT) due to hyperacute ischemic stroke being easily diagnosed on an MRI. LVO can be assessed via magnetic resonance angiography (MRA) without the use of a contrast agent. Although a previous study has reported favorable interdevice agreement between smartphone applications and desktop PC monitoring systems for evaluating CT images [8] and diffusion-weighted imaging on MRI [9], interdevice agreement for LVO on MRI is unknown. This study compares the speed and accuracy of LVO diagnosis by vascular neurologists between the JOIN smartphone application (Smartphone-LVO) and a conventional hospital-based image viewer using a desktop PC monitor (PC-LVO).

## Methods

### Patients

Consecutive patients admitted with ischemic stroke to Jikei University Hospital, Japan, between January 2016 and September 2017 were retrospectively enrolled in the study. The subtype of ischemic stroke was classified according to the TOAST (Trial of ORG 10172 in Acute Stroke Treatment) [10]. Stroke severity on admission was assessed using the National Institutes of Health Stroke Scale score [11]. After reviewing patients’ medical records, 108 patients with ischemic stroke in the middle cerebral artery territory identified on MRI within 24 hours of onset were enrolled in the study. Patients who did not undergo MRI upon admission or for whom imaging was not available were excluded from the study.

### JOIN Smartphone Application

The JOIN (Allm, Tokyo, Japan) smartphone application enables teleradiology of medical images, including MRI, CT, ultrasonography, and angiography (Figure 1). Using JOIN, medical information can be shared immediately among the stroke team via SMS. Images from the initial MRI brain examination are sent immediately from the picture archiving and communication system (PACS) on the hospital server to each smartphone on which JOIN is installed. Users can enlarge and evaluate the images through a simple touch sequence on the screen of their smartphone.

### MRI Protocols

All patients underwent routine 3D time-of-flight (TOF) MRA of the intracranial arteries and fluid-attenuated inversion recovery (FLAIR) of the brain on a 1.5T scanner (MAGNETOM Avanto and MAGNETOM Symphony). The imaging parameters were TOF MRA: repetition time (TR)/ echo time (TE) = 23/7.15 ms, flip angle = 20°, section thickness = 0.6 mm, matrix = 320 × 288, field-of-view (FOV) = 18 cm; FLAIR: TR/TE = 9000/94 ms, flip angle = 160°, section thickness = 5 mm, section gap = 1.5 mm, matrix = 256 × 256, FOV = 21 cm.

### Image Review

**Figure 2:**
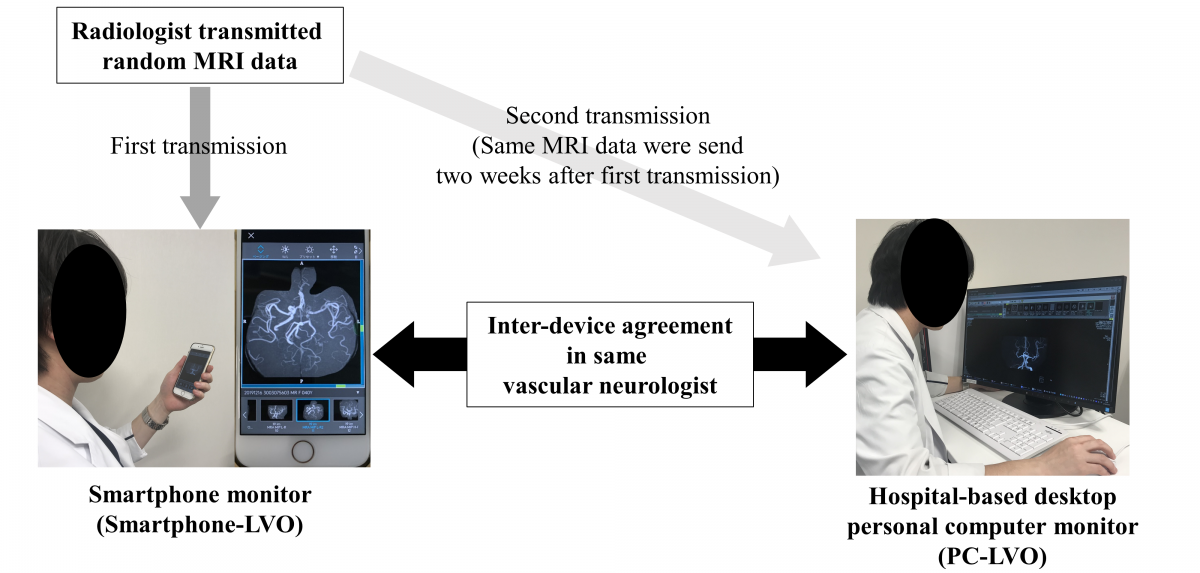
Flow diagram of the study design. LVO: large vessel occlusion, MRI: magnetic resonance imaging.

The image review process is shown in Figure 2. Prior to the review, 2 readers (TK and KS, Readers 1 and 2 with 8 years and 10 years of experience in vascular neurology, respectively) installed JOIN on a Jikei University Hospital iPhone (version 7; Apple). After they confirmed the operation of the application, a radiologist transmitted the MRA and FLAIR data to the application. Patient image sets were presented randomly, and the readers were blinded to all background and clinical information. Both readers evaluated all the images from all patients. After receiving the MRA and FLAIR images, the readers independently assessed them on the JOIN application for the presence or absence of intracranial arterial occlusion; in the case of LVO, the site of arterial occlusion was identified. In patients with acute stroke, it is important to check for hyperintense vessel sign (HVS) on FLAIR, which suggests the presence of LVO [12]. HVS occurs because of slow flow due to LVO but is not an indicator of infarction. Therefore, the 2 readers evaluated the MRA images for LVO and evaluated the FLAIR images for HVS. The arterial occlusion site was classified as internal carotid artery (ICA), the horizontal segment of the middle cerebral artery (M1), or insular or cortical segments of the middle cerebral artery (M2 or M3, respectively). The diagnosis accuracy of arterial occlusion was defined as interdevice variability between Smartphone-LVO and PC-LVO. We recorded the interpretation time (minutes), defined as the time between receiving the MRI data to completing the interpretation. We used 1 to 4 min to interpret times in segments of 0 to 1 min, 1 to 2 min, 2 to 3 min, and 3 to 4 min. The 2 readers evaluated all MRI data using the hospital desktop PC monitor in the same manner 2 weeks later.

### Statistical Analysis

Interdevice agreement for evaluating the presence of arterial occlusion was analyzed between Smartphone-LVO and PC-LVO via kappa statistics. Kappa scores for agreement were rated as follows: poor (<0.20), fair (0.21-0.40), moderate (0.41-0.60), favorable (0.61-0.80), almost perfect (0.81-1.0).

Wilcoxon signed-rank test was used to compare image interpretation times between Smartphone-LVO and PC-LVO. 

*P* values <.05 indicated statistical significance. All analyses were performed using SPSS for Windows (version 22.0; BM-Armonk).

### Standard Protocol Approvals and Registrations

The Regional Ethics and Hospital Management Committee of the Jikei University School of Medicine approved the study (approval No. 29-197). The board waived the need for patient consent by giving patients from whom data had been collected the opportunity to opt-out of the study.

## Results

### Patient Characteristics

Table 1 lists the patient characteristics. We enrolled 108 patients with a median age of 69 years and a median NIHSS score of 4 upon admission, of whom 72 (66%) were male.

**Table 1:** Patient characteristics.

Variable		All patients (n=108)
Age (years), median (IQR)		69 (61-80)
Sex (male), n (%)		72 (67)
NIHSS^a^ score on admission, median (IQR)		4 (2-7)
**TOAST^b^ classification, n (%)**		
	Large-artery atherosclerosis	8 (7)
	Small-vessel occlusion	14 (13)
	Cardio-embolism	35 (32)
	Other determined etiology	9 (8)
	Undetermined	42 (39)
Modified Rankin scale at 3 months, median (IQR)		1 (1-3)
MRI^c^ time from onset (minutes), median (IQR)		244 (107-623)

^a^NIHSS: National Institute Health Stroke Scale.

^b^TOAST: Trial of ORG 10172 in Acute Stroke Treatment.

^c^MRI: magnetic resonance imaging.

### Interdevice Agreement Between Smartphone-LVO and PC-LVO

**Figure 3:**
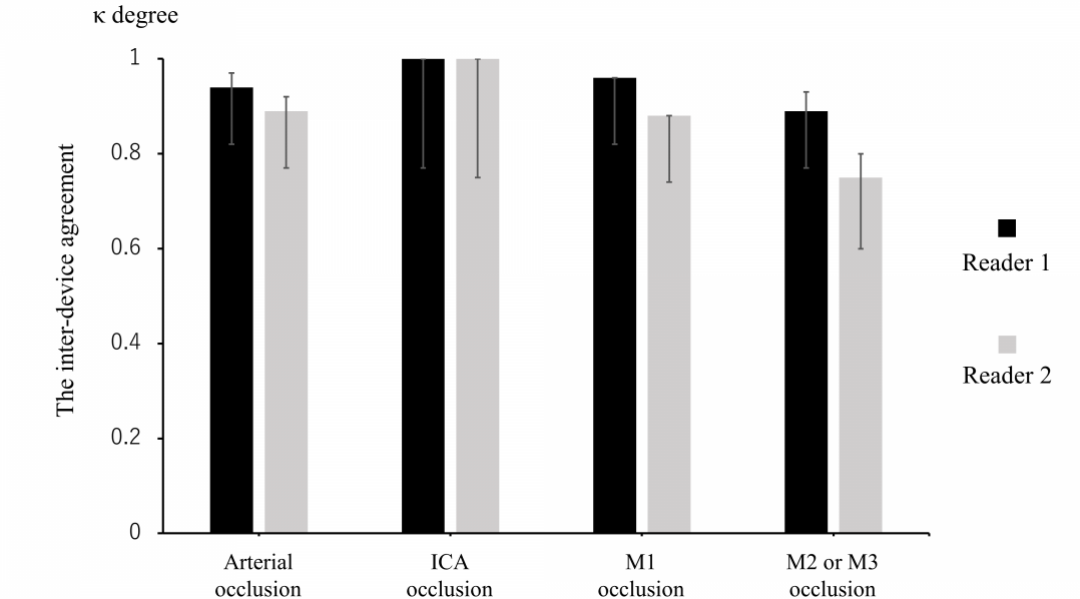
Interdevice agreement for evaluation of intracerebral arterial occlusion and for the 3 vessel categories. The degree of interdevice agreement was almost perfect in the evaluation of the arterial occlusion sites and was higher for proximal than distal arterial occlusion sites. ICA: internal carotid artery.

Interdevice agreement for evaluating the presence or absence of intracranial arterial occlusion was almost perfect between Smartphone-LVO and PC-LVO (Reader 1: κ=0.94, 95% CI 0.82-0.97; *P*<.001 and Reader 2: κ=0.89, 95% CI 0.77-0.92; *P*<.001; Table 2; Figure 3). Regarding detection of lesions in individual arteries, interdevice agreement was almost perfect for ICA and M1 occlusion (Tables 3 and 4; Figure 3). For analyzing M2 or M3 occlusion, interdevice agreement by Reader 1 was almost perfect (κ=0.89, 95% CI 0.77-0.93; *P*<.001,) and that by Reader 2 was favorable (κ=0.75, 95% CI 0.60-0.80 *P*<.001; Table 5; Figure 3).

**Table 2:** Comparison of evaluation of the presence or absence of intracranial arterial occlusion between Smartphone-LVO and PC-LVO.

	Smartphone-LVO^a^	PC-LVO	
		Occlusion	No occlusion
**Reader 1 (TK)**			
	Occlusion	33	2
	No occlusion	1	72
**Reader 2 (KS)**			
	Occlusion	42	5
	No occlusion	1	60

^a^LVO: large vessel occlusion.

**Table 3:** Comparison of identification of internal carotid artery occlusion between Smartphone-LVO and PC-LVO.

	Smartphone-LVO^a^	PC-LVO	
		Occlusion	No occlusion
**Reader 1 (TK)**			
	Occlusion	8	0
	No occlusion	0	100
**Reader 2 (KS)**			
	Occlusion	7	0
	No occlusion	0	101

^a^LVO: large vessel occlusion.

**Table 4:** Comparison of identification of horizontal segment of middle cerebral artery occlusion between Smartphone-LVO and PC-LVO.

	Smartphone-LVO^a^	PC-LVO	
		Occlusion	No occlusion
**Reader 1 (TK)**			
	Occlusion	16	1
	No occlusion	0	91
**Reader 2 (KS)**			
	Occlusion	19	0
	No occlusion	4	85

^a^LVO: large vessel occlusion.

**Table 5:** Comparison of identification of insular or cortical segments of middle cerebral artery occlusion between Smartphone-LVO and PC-LVO.

	Smartphone-LVO^a^	PC-LVO	
		Occlusion	No occlusion
**Reader 1 (TK)**			
	Occlusion	31	4
	No occlusion	1	72
**Reader 2 (KS)**			
	Occlusion	34	11
	No occlusion	2	61

^a^LVO: large vessel occlusion.

### Interpretation Time Between Smartphone-LVO and PC-LVO

**Figure 4:**
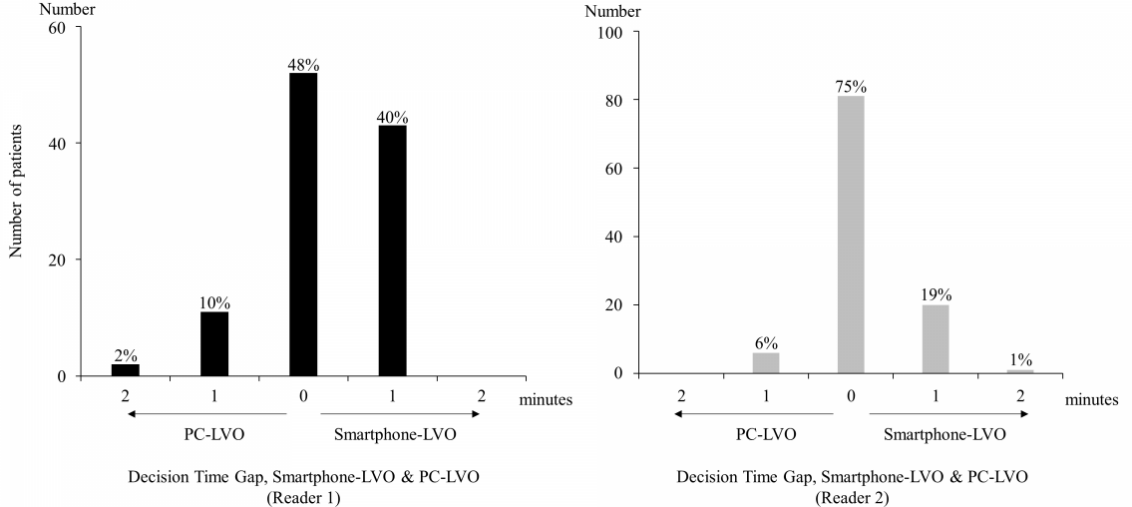
Difference in interpretation times between Smartphone-LVO and PC-LVO for Readers 1 and 2. The difference in interpretation time between Smartphone-LVO and PC-LVO was <1 min in 98% of all patient datasets. Reader 1, left; Reader 2, right. LVO: large vessel occlusion.

Wilcoxon signed-rank test (2-tailed) revealed that the interpretation time was significantly longer for Smartphone-LVO than PC-LVO (Reader 1: z=–3.547; *P*<.001 and Reader 2: z=–2.921; *P*=.003). However, the median interpretation time was the same between Smartphone-LVO and PC-LVO for each reader (Reader 1: 2 min for each method and Reader 2: 1 min for each method; Table 6). The interpretation time difference between Smartphone-LVO and PC-LVO was less than 1 min in 98% of the patient data sets in our series (Figure 4).

**Table 6:** Comparison of interpretation times between Smartphone-LVO and PC-LVO.

			Interpretation time of Smartphone-LVO^a^	Interpretation time of PC-LVO
**Reader 1 (TK)**				
	Median minute (IQR)	2(2-2)		2(1-2)
	Standard deviation	0.64		0.68
	Min minute	1		1
	Max minute	4		3
**Reader 2 (KS)**				
	Median minute (IQR)	1(1-2)		1(1-1)
	Standard deviation	0.49		0.34
	Min minute	1		1
	Max minute	3		2

^a^LVO: large vessel occlusion.

## Discussion

### Principal Findings

The study results show that the smartphone application yielded an accurate diagnosis of anterior intracranial arterial occlusion in times similar to diagnoses completed using the hospital-based desktop PC monitor. Several studies have reported the use of smartphone applications for the management of stroke [4,13-15]; however, no study has reported their use for assessing LVO. To the best of our knowledge, this is the first article to examine interdevice agreement and compare interpretation times for MRA and HVS on FLAIR imaging between a smartphone application and a hospital desktop PC monitor.

The JOIN smartphone application shows promise for use in acute stroke management for several reasons. JOIN provides a platform for simultaneously sharing important clinical information among the entire stroke team via PACS, whether on duty or on call. Because the information shared within the team is secured, JOIN can seamlessly support intrahospital teleradiology for MT in the “drip and ship” acute stroke management model. Conventionally, after receiving a stroke call, the vascular neurologist must immediately access a desktop PC and log into the network and server before browsing images sent via PACS. The neurologist’s interpretation of the neuroimaging examinations is then shared over the telephone with one person in the stroke team. JOIN can substantially improve the speed of this process by reducing the time taken to evaluate neuroimaging, particularly in situations where the neurologist is not near a desktop PC or is not at the hospital at the time that treatment for hyperacute stroke is being decided. The utilization of the JOIN application should simplify and shorten the MT decision-making process.

Regarding the telestroke system, while peer-to-peer network protocols are standard, they are limited in that the sender communicates only with one receiver in the stroke team, much like a closed circuit loop. In contrast, the JOIN application enables multicasting communications from one sender to many receivers. The team leader pre-authorizes which stroke team members participate in the secure group chat, and all of the patient’s data are centralized to the group chat. The entire stroke team can then receive notifications of the stroke patient’s arrival, view neuroimaging examinations, and review the medical plan via JOIN. Thus, important information is shared immediately among all members. JOIN also enables messaging among the team and displays neuroimaging files at a high resolution. Because the decision-making process is tracked via messages displayed on JOIN, the application plays a role in avoiding errors. Thus, combining the conventional telestroke system with JOIN would be desirable for arranging hyperacute stroke care in a complex and time-sensitive environment.

Finally, the telestroke system, comprising the 3 components of teleconsultation (doctor to patient), teleconferencing, and teleradiology, is confronted with developmental challenges in Japan. It is difficult for hospitals in Japan to introduce the real-time interactive video conference systems required for tele-examination using a hub and spoke model because there is no legal or regulatory provision for doctors on the hub side regarding patient examinations or a provision for medical decisions [16]. In addition, the Japanese government only approves medical practice via conventional encounters with a unique patient in emergency settings. Therefore, teleconsultation is only allowed under strict conditions (ie, for monitoring chronic disease after a second visit). In contrast, teleradiology is commonly used in Japan because of national insurance coverage. The advantages of using JOIN for acute stroke care include its portability, quick accessibility of target neuroimages, and its financial viability. In the near future, the Japanese Stroke Association plans to expand their practical guidelines for telestroke to include ICT solutions, under which JOIN would be considered for use.

### Limitations

Our study has several limitations. First, the MRA images were evaluated by only 2 vascular neurologists, both of whom were experienced. In a future study, more evaluators should be used, including less experienced evaluators. Second, the study did not directly prove that the smartphone application was useful for early reperfusion therapy. Finally, the study only evaluated anterior circulation ischemic stroke; its applicability to posterior circulation ischemic stroke has yet to be investigated.

### Conclusions

A smartphone application yielded an accurate diagnosis of anterior intracranial arterial occlusion in times similar to diagnoses completed using the hospital-based desktop PC monitor. It also enabled access to all patient information regardless of the user’s location. Thus, this system can play an important role in organizing the stroke team to manage patients with hyperacute ischemic stroke.

The authors declare that all supporting data are available within the article.

## Abbreviations

FLAIR: fluid-attenuated inversion-recovery
FOV: field-of-view
HVS: hyperintense vessel sign
ICA: internal carotid artery
ICT: information and communication technology
IV-tPA: intravenous tissue plasminogen activator
LVO: large vessel occlusion
MRA: magnetic resonance angiography
MRI: magnetic resonance imaging
MT: mechanical thrombectomy
PACS: picture archiving and communication system
TE: echo time
TOF: time-of-flight
TR: repetition time
